# Acting on climate change and health in Victoria

**DOI:** 10.5694/mja2.50527

**Published:** 2020-03-13

**Authors:** Brett Sutton, Vanora Mulvenna, Daniel Voronoff, Tiernan Humphrys

**Affiliations:** ^1^ Department of Health and Human Services State Government of Victoria Melbourne VIC

**Keywords:** Climate change, Public health, Public policy

*Victorian legislation will help the health sector reduce emissions and adapt to climate change*

Victoria is taking decisive action to address the significant risks posed by climate change. In part, the *Climate Change Act 2017* (Vic) requires the Victorian government to:

- contribute to whole‐of‐government emissions reduction to meet net zero emissions by 2050;
- prepare whole‐of‐government and sector emission reduction pledges;
- develop and implement adaptation action plans, detailing the risks and impacts on systems (including health and human services); and
- endeavour to ensure that any decision made by the Victorian government and any policy, program or process developed or implemented takes account of climate change.

Victoria is one of four Australian states and territories, along with the Australian Capital Territory, Tasmania and South Australia, with emission reduction targets specified in legislation. After the ACT, which has a target of net zero emissions by 2045, Victoria has the next most ambitious target prescribed in legislation.^1^

Importantly, the Climate Change Act requires decision makers to have regard to climate change in their administration of other legislation scheduled under the Act. For example, the requirement to prepare state and municipal public health and wellbeing plans under the *Public Health and Wellbeing Act 2008* (Vic) has been scheduled under the Climate Change Act for this purpose.

## The critical role of public health in driving action on climate change

Public health is focused on protecting and promoting the health of the whole population, and preventing illness, injury and disability.^2^ The climate crisis is a fundamental threat to public health, and Victoria is experiencing significant impacts from events which are becoming more frequent and intense due to climate change. For example:

- the 2009 Victorian heatwave resulted in 374 excess estimated deaths and a 12% increase in public hospital emergency department presentations (compared with the 5‐year average);^3^
- the 2014 Victorian heatwave resulted in 167 excess estimated deaths, and resulted in a fivefold increase in heat‐related public hospital emergency department presentations compared with what was expected;^4^ and
- following the 2016–2017 Victorian floods, there was a large increase in mosquitos, which resulted in a 7.5‐fold increase in Ross River virus disease compared with the previous year.^5^

Further, the 2019–20 bushfires in Australia, which were preceded by record springtime fire danger weather,^6^ caused hazardous air quality in population centres, bringing health concerns from smoke exposure into sharp focus, in addition to the tragic direct loss of life. These and other events highlight the central role of public health in driving action on climate change, seeking solutions and supporting the community to adapt to its impacts.

In August 2019, the Victorian Department of Health and Human Services published a public health and wellbeing plan for 2019–2023.^7^ The plan recognises that climate and environmental conditions have a profound influence on a population's health. The growing threats to health from our changing climate, therefore, need to be central to our thinking and actions into the future.

Climate change is included as one of four key focus areas in the new plan, which aims to achieve resilient and safe communities that are adapting to the public health impacts of climate change, decreased health impacts associated with climate change, and increased action to reduce greenhouse gas emissions and realise health co‐benefits.^7^

The new plan also continues to recognise the current pressing problems of public health including obesity, harms from tobacco, and our sedentary lifestyle. Continued action in these areas will both improve the health and wellbeing of Victorians and reduce downstream health system costs and environmental impacts. For example, in 2018–19 every occupied bed‐day in a Victorian public hospital generated on average 120 kg of carbon dioxide equivalents and 3.65 kg of waste, and used 630 L of water.^8^ As a result, investment in initiatives to reduce rates of preventable diseases will also contribute to emissions reductions in the health sector.

## Victoria's health and human services climate change strategy

More broadly, the Victorian Department of Health and Human Services is undertaking comprehensive climate change planning and is currently preparing a climate change strategy, which comprises an emissions reduction plan and an adaptation action plan.

The emissions reduction plan is focused on reducing greenhouse gas emissions from the department, and health and human service funded agencies (including public hospitals, ambulance services, public housing and departmental buildings). It will provide an important contribution to whole‐of‐government emissions reduction targets and will be finalised in its first iteration following the Victorian government's planned announcement of interim emission reduction targets in 2020.

The department's first adaptation action plan outlines the risks to public health, and health and human services, and identifies actions to address those risks. Responding to the urgent need for action, the plan is being implemented in advance of statutory requirements, which come into force in 2021.^9^

Together, these plans will form an integrated climate change strategy, recognising that action in both these domains is urgently needed to protect the health and wellbeing of Victorians from the climate crisis.

## The role of the Victorian health sector in climate change mitigation and adaptation

If the global health sector were a country, it would be the fifth‐largest greenhouse gas emitter on the planet. It is estimated that the sector's carbon footprint is equivalent to 4.4% of global net emissions.^10^ Given this, the health sector has an important role to play in showing leadership on this critical health issue. The Environmental Sustainability Strategy 2018–19 to 2022–23 sets the direction for Victorian health services to improve their environmental performance and adapt to climate change.^11^

In 2018, the Department of Health and Human Services completed a climate risk assessment of all public health infrastructure to better understand climate risk to the system and specific health assets (unpublished report). The assessment identified that in a scenario of 4° warming at 2100, riverine flooding, coastal inundation, bushfire, extreme wind, soil movement and extreme heat would pose risks to public health infrastructure. The findings are being incorporated into capital and engineering guidelines to ensure the design and delivery of new health infrastructure will be able to operate in our future climate.^9^

Since 2015–16, the Greener Government Buildings program has allocated $31 million to invest in energy efficiency and solar power in public health facilities.^11^ These projects aim to deliver cumulative emissions savings of around 117 000 tonnes to 2026. The department has a range of programs to reduce the carbon intensity and emissions of the health sector. It requires all public hospitals and health services to have an environmental management plan and report publicly on their environmental performance. It also administers energy and waste programs and develops technical resources, such as guidance on vehicle fleet efficiency, to support action at the local level.^12^

## Conclusion

Climate change represents an existential threat to human health. The health sector, as a key support to human health and wellbeing, as well as a significant contributor to Australia's emissions, has a key role to play in addressing this threat.

Victoria has set a foundation for fundamental change in legislation. The supporting policy and strategy elements, focused on population health and wellbeing, are additional enablers for such change. We have an opportunity to build a more resilient sector while limiting our contribution to the climate crisis. We must take it.

## Competing interests

All authors are Victorian state government employees.

## Provenance

Commissioned; externally peer reviewed.
